# Subjective Discomfort of TMS Predicts Reaction Times Differences in Published Studies

**DOI:** 10.3389/fpsyg.2018.01989

**Published:** 2018-10-18

**Authors:** Nicholas Paul Holmes, Lotte Meteyard

**Affiliations:** ^1^School of Psychology, University of Nottingham, Nottingham, United Kingdom; ^2^School of Psychology and Clinical Language Sciences, University of Reading, Reading, United Kingdom

**Keywords:** TMS, transcranial magnetic stimulation, side-effects, artifact, reaction times, posterior parietal cortex, inferior frontal gyrus, anterior temporal lobe

Transcranial magnetic stimulation (TMS) was developed 30 years ago, in part to decrease the peripheral side-effects associated with transcranial electrical stimulation (Barker, 1991). TMS has been effective in that aim, and great advances have been made over the past 30 years. TMS can still be uncomfortable and painful, however, as it stimulates excitable superficial tissue including scalp muscles and peripheral nerves (Maizey et al., 2013). This causes annoyance, pain, and muscle twitches (i.e., discomfort) that vary systematically across the scalp (Meteyard and Holmes, 2018). While superior and posterior scalp locations are associated with almost no discomfort, inferior frontal and temporal locations are associated with significant discomfort. This discomfort can include sharp pain and strong contractions of scalp, head, and neck muscles. In protocols where TMS and a behavioral task are separated by time (“off-line”), these peripheral side-effects of brain stimulation may not affect subsequent task performance. But, in protocols where TMS is applied simultaneously with the behavioral task (“on-line”), these side-effects of TMS might interfere significantly with performance.

Meteyard and Holmes (2018) found that participants' subjective ratings of the annoyance, pain, and muscle twitches caused by single pulse TMS was significantly and strongly correlated with changes in reaction time (RT) on two simple stimulus-response congruency tasks. Ratings of muscle twitches accounted for 43% of the variance in RT. TMS over parietal areas (e.g., P3/P4 electrode locations) resulted in a 9 ms *decrease* in RT. TMS over inferior locations (e.g., anterior temporal lobe) led to RT *increases* as large as 81ms. Thus, TMS-related peripheral side effects must be taken into account when studying the effects of on-line TMS. In particular, when effects of TMS are compared with a no TMS or sham condition, or when two TMS locations are compared, researchers need to control for differences in TMS-related discomfort.

For this Opinion, we investigated whether the TMS-related discomfort measured in our previous work could predict the reported differences in RT in studies published in the last 10 years. We searched for studies using on-line single-pulse TMS over the least uncomfortable (superior parietal) and most uncomfortable (anterior temporal, inferior frontal) brain areas. PubMed was searched with the query “(((TMS OR (transcranial magnetic stimulation)) AND parietal)) AND (“2008/01/01” [Date-Publication]:“3000” [Date-Publication]) AND (reaction OR response).” A second and third search replaced the term “parietal” with “anterior temporal” and “inferior frontal.” Three hundred and seven results were returned for parietal, 14 for anterior temporal (1 more after extending the search to 20 years) and 65 for inferior frontal. Studies were included if we could access the full text, they were in English, studied healthy participants, and used on-line single-pulse TMS. We excluded studies using repetitive TMS, reporting only clinical data, or not containing RTs from a behavioral task. The parietal search focused on superior parietal or intraparietal locations; excluding more inferior supramarginal or angular gyri targets. Seventeen studies met the inclusion criteria (15 parietal, 2 inferior frontal). To increase the sample size, we relaxed the inclusion criteria to include double-, and triple-pulse on-line TMS studies (total: 22 parietal, 6 inferior frontal, 2 anterior temporal).

From these articles, we extracted all the locations stimulated (including any additional non-parietal or non-frontal/temporal areas) and the RTs associated with those locations, averaging across other conditions. These reported locations were matched as closely as possible with those stimulated in Meteyard and Holmes (2018), which mostly corresponded to 10–10 EEG electrode locations. As predictor variables, we used the mean of median rating of muscle twitches and the mean effect of TMS on RT (i.e., change in RT with TMS as compared to no TMS), which were extracted from http://www.tms-smart.info. For the outcome variable, within each study, differences between RTs for each location and a control condition (no TMS, sham TMS, TMS over vertex, or the average of these) were calculated. Each study contributed at least one data point (mean ± *SD* = 1.6 ± 1.1 data points per study, both for the 17 single-pulse studies and across all 30 studies). Where multiple TMS locations, groups, or experiments were reported, each study contributed multiple data points. RT differences and standardized RT differences (RT difference/pooled SD) were correlated with the per-location mean of median ratings of muscle twitches and effect of TMS on RT from Meteyard and Holmes (2018). 95% confidence intervals for *r*-values were obtained by bootstrapping over 10,000 iterations. Results are plotted in Figure 1. Full data, analysis scripts, and supplementary figures can be found at https://osf.io/f49vn/, in Supplementary Table 1, and at https://tms-smart.info.

**Figure 1:**
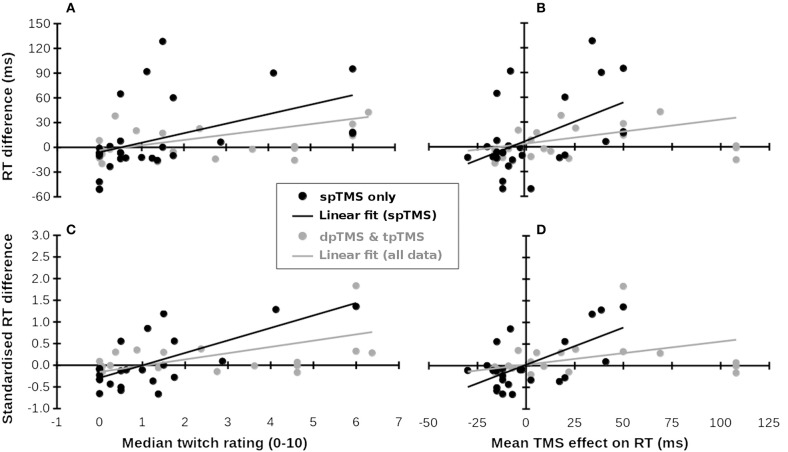
Subjective ratings of muscle twitches and TMS-related RT increases (x-axes, from Meteyard and Holmes, 2018) predict RT differences in published studies (y-axes, multiple studies). **(A–D)** Show different measures. Black symbols and lines: single-pulse TMS data; Gray symbols: Double- (dp) and triple-pulse (tp) TMS data. Gray lines: Linear fit across all available data. Data for the x-coordinates were taken from Meteyard and Holmes (2018). Data for the y-coordinates were taken from Baldassarre et al. (2016), Busan et al. (2009a,b,c); Busan et al. (2012), Cattaneo et al. (2009, 2014), Chica et al. (2011), Fautrelle et al. (2013), Jackson et al. (2015), Johnson et al. (2012), Kehrer et al. (2015), Koch et al. (2008), Ku et al. (2015), Machizawa et al. (2010), Newman-Norlund et al. (2010), Oshio et al. (2010), Pasalar et al. (2010), Renzi et al. (2011), Ricci et al. (2012), Salillas et al. (2009, 2012), Schuhmann et al. (2009), Shinshi et al. (2015), Teige et al. (2018), Tunik et al. (2008), Vernet et al. (2008), Wheat et al. (2013), and Yan et al. (2016). **(A)** Median twitch ratings (x-axis) predict raw RT differences (y-axis). **(B)** Mean TMS effect on RT predicts raw RT differences. **(C)** Median twitch ratings (x-axis) predict standardized RT differences (y-axis). **(D)** Mean TMS effect on RT predicts standardized RT differences.

For single-pulse TMS studies (28 samples), differences in RT between TMS and control conditions were significantly correlated with both the mean of median ratings of muscle twitches, *r*_26_ = 0.473, 95%CI = {0.209, 0.757}, *p* = 0.011 (Figure 1A), and the mean effect of TMS on RT from Meteyard and Holmes (2018), *r*_26_ = 0.497 {0.173, 0.740}, *p* = 0.007 (Figure 1B). For each increment in twitch rating, RT increased by 11.5 ms (*cf*. 15 ms in Meteyard and Holmes, 2018). This relationship strengthened when the observed RT data were standardized, both for twitches, *r*_24_ = 0.690 {0.265, 0.872}, *p* < 0.001 (Figure 1C), and RTs, *r*_24_ = 0.624 {0.165, 0.846}, *p* = 0.001 (Figure 1D). Each increment in twitch rating was associated with a change of 0.29SD in RT. Expanding the dataset to include double- and triple-pulse online TMS studies (Figure 1, gray symbols) weakened these relationships (twitches & raw RT, *r*_47_ = 0.365 {0.141, 0.597}, *p* = 0.01; RT & raw RT, *r*_47_ = 0.272 {0.066, 0.530}, *p* = 0.06; twitches & standardized RT, *r*_41_ = 0.529 {0.245, 0.727}, *p* < 0.001; RT & standardized RT, *r*_41_ = 0.346 {0.138, 0.613}, *p* = 0.023). TMS over superior parietal cortex, and over other scalp regions where little discomfort is felt, results in a small *decrease* in RT (bottom-left of Figures 1A–D), whereas TMS at scalp locations causing significant discomfort generally increases RT (top-right of Figures 1A–D). In all analyses, the bootstrapped 95% confidence intervals for the correlation coefficients did not include 0. In an additional check for robustness of the correlations, each dataset was submitted to 10,000 iterations of a “leave N out” analysis. On each iteration, the original dataset was re-analyzed, each time leaving out a randomly-selected N datapoints, where N varied between 1 and 20. The 95% confidence interval for all 8 *r*-values reported above included *r* = 0 only when at least 13 datapoints had been left out of the analysis. These analyses suggest that the observed correlation is positive and does not depend on particular datapoints being included.

Subjective ratings of muscle twitch strength can predict the effects of TMS on RT at a range of scalp locations, across a range of tasks, and across a range of TMS protocols. Two qualifications should be made. First, the reported correlations may be a slight underestimation of the true effect. Across studies, the TMS intensity used (maximum stimulator output) was inversely-related both to predicted discomfort [*r*_(28)_ = −0.507, *p* = 0.004], and to predicted RT changes associated with stimulation at that site [from Meteyard and Holmes, 2018 data, *r*_(28)_ = −0.524, *p* = 0.003], although this was not significant for the RT differences reported by the studies themselves [*r*_(26)_ = −0.124, *p* = 0.55]. This could be due to the finding that higher discomfort is associated with lower scalp-to-cortex distances (Meteyard and Holmes, 2018), thus requiring lower TMS intensity. Or, it could be that researchers use lower TMS intensities when their participants report greater discomfort. Second, the reported correlations were weaker when the double- and triple-pulse TMS studies' data were included. This could be due to genuine differences in discomfort between single-pulse and multi-pulse TMS, or to the greater heterogeneity of study types and TMS parameters when all TMS protocols were included.

We cannot conclude that the results of the reviewed studies merely reflect differences in the peripheral side-effects of TMS, since we deliberately averaged across experimental conditions (but not scalp locations) within each study. Instead, we can conclude that RTs under TMS differ systematically across scalp locations (i.e., not necessarily caused by differences in, or the effects of TMS on, the underlying brain regions). Direct comparisons of RT between TMS at different scalp locations are confounded by differences in these peripheral side-effects, and must be interpreted with caution.

The data from Meteyard and Holmes (2018) has been used to create interactive maps available at http://www.tms-smart.info. Researchers can use these interactive maps to select control sites that may help account for TMS-related discomfort. In the next 30 years of studies using TMS, we recommend systematically controlling for the side-effects associated with magnetic stimulation of the scalp, for example by choosing control sites with similar levels of discomfort.

## Author contributions

All authors listed have made a substantial, direct and intellectual contribution to the work, and approved it for publication.

### Conflict of interest statement

The authors declare that the research was conducted in the absence of any commercial or financial relationships that could be construed as a potential conflict of interest.
